# Results from a proactive follow-up intervention to improve linkage and retention among people living with HIV in Uganda: a pre-/post- study

**DOI:** 10.1186/s12913-018-3735-0

**Published:** 2018-12-06

**Authors:** Caroline E. Boeke, Vennie Nabitaka, Andrea Rowan, Katherine Guerra, Pamela Nawaggi, Vivienne Mulema, Victor Bigira, Eleanor Magongo, Patricia Mucheri, Andrew Musoke, Cordelia Katureebe

**Affiliations:** 1 Clinton Health Access Initiative (CHAI), 383 Dorchester Road, Suite 400, Boston, MA 02127 USA; 2 CHAI, Kampala, Uganda; 3 Ministry of Health, Kampala, Uganda

**Keywords:** Linkage to care, Retention in care, Follow-up, Quality of care, Uganda, Africa

## Abstract

**Background:**

Despite gains in HIV testing and treatment access in sub-Saharan Africa, patient attrition from care remains a problem. Evidence is needed of real-world implementation of low-cost, scalable, and sustainable solutions to reduce attrition. We hypothesized that more proactive patient follow-up and enhanced counseling by health facilities would improve patient linkage and retention.

**Methods:**

At 20 health facilities in Central Uganda, we implemented a quality of care improvement intervention package that included training lay health workers in best practices for patient follow-up and counseling, including improved appointment recordkeeping, phone calls and home visits to lost patients, and enhanced adherence counseling strategies; and strengthening oversight of these processes. We compared patient linkage to and retention in HIV care in the 9 months before implementation of the intervention to the 9 months after implementation. Data were obtained from facility-based registers and files and analysed using multivariable logistic regression.

**Results:**

Among 1900 patients testing HIV-positive during the study period, there was not a statistically significant increase in linkage to care after implementing the intervention (52.9% versus 54.9%, *p* = 0.63). However, among 1356 patients initiating antiretroviral therapy during the follow-up period, there were statistically significant increases in patient adherence to appointment schedules (44.5% versus 55.2%, *p* = 0.01) after the intervention. There was a small increase in Ministry of Health-defined retention in care (71.7% versus 75.7%, *p* = 0.12); when data from the period of intervention ramp-up was dropped, this increase became statistically significant (71.7% versus 77.6%, *p* = 0.01). The increase in retention was more dramatic for patients under age 19 years (*N* = 84; 64.0% versus 83.9%, *p* = 0.01). The cost per additional patient retained in care was $47.

**Conclusions:**

Improving patient tracking and counseling practices was relatively low cost and enhanced patient retention in care, particularly for pediatric and adolescent patients. This approach should be considered for scale-up in Uganda and elsewhere. However, no impact was seen in improved patient linkage to care with this proactive follow-up intervention.

**Trial registration:**

Pan African Clinical Trial Registry #PACTR201611001756166. Registered August 31, 2016.

## Background

Ensuring that people living with HIV (PLWHIV) are enrolled in care and remain on treatment will be essential to reducing the global burden of HIV. However, in Uganda and elsewhere in sub-Saharan Africa, patient attrition in the testing and treatment cascade remains a persistent challenge [1–5]. A number of patient-level factors such as younger age, lower socioeconomic status, residence in rural areas, poorer health at diagnosis/treatment initiation, and experience of stigma have been found to be related to greater patient attrition in previous studies [6–11]. However, facility-level practices may play a role as well. Recent systematic reviews have identified several types of facility-level interventions that have been shown in certain settings to increase patient linkage and reduce patient attrition such as point-of-care testing [12–14], integrated services [12, 15], task-shifting care to laypeople or lower cadre health workers [14–17], service improvements/enhanced clinic operations [12], family-centered services [15], better counseling and education to provide adherence support [16], SMS messaging to support patient adherence to treatment [16], and differentiated care among stable patients [17, 18]. Despite the success of these interventions in particular settings, more examples are needed of successful real-world implementation of low-cost, scalable solutions in national HIV programs. Moreover, despite greater attrition in children and adolescents compared to adults [19, 20], there is currently very limited data available on effective interventions to improve linkage and retention in adolescent patients [17].

In this study, we implemented a strengthened management intervention for proactive patient follow-up and enhanced counseling at 20 government-supported facilities in Central Uganda with high attrition from HIV care. We set out to evaluate linkage to care among individuals testing positive for HIV and six-month retention in care in antiretrovial therapy (ART) patients before versus after rollout of the intervention. At baseline, we noted that linkage to care was quite low (approximately 50%) and that adolescents, females, and patients in more rural settings had lower linkage and retention [21]. This study provides a comprehensive assessment of a holistic but relatively low-cost intervention to improve outcomes in PLWHIV across age groups.

## Methods

### Study setting

This study was implemented in 20 randomly selected facilities across 14 districts in Central Uganda meeting the following criteria: Offered ART to pediatric and adult patients starting January 1, 2015 or earlier, low estimated annual retention among patients on ART (approximately 35–75% in 2015 using national District Health Information System (DHIS) 2 estimates), and high ART patient volume (> 120 patients enrolled in 2015 according to DHIS 2). Of the 27 eligible facilities, those that were primarily mental health facilities (*n* = 1), had related interventions in place (*n* = 1), or were located on islands (*n* = 4) were excluded, and of the 21 remaining facilities, 20 were randomly selected. All eligible facilities were level III or IV and all selected facilities agreed to participate. Level III and IV health facilities are primary health care facilities with general outpatient clinics and maternity wards. Level IV facilities also run operating theatres offering cesarean sections and minor surgeries.

### Description of the intervention

The intervention included several components: 1) Training of expert clients (PLWHIV working at the facility as lay staff) in best practices for patient linkage/follow-up, including provision of flip charts with concrete guidance on best practices for patient follow-up and counseling and clearly defined roles and responsibilities; 2) Training and empowerment of facility or ART in-charges in best practices for supervision of patient linkage/follow-up, including provision of a trainer’s manual on best practices for patient follow-up and counseling; 3) Training of district staff in enhanced supervision and oversight of patient follow-up at facilities through monitoring and stronger inter-facility communication, including provision of a trainer’s manual on best practices for patient follow-up and counseling; 4) Financial and logistical support to facilities: facilities were provided with a cellular telephone and a small amount (approximately $72 USD/month) of funding for phone follow-up and home visits. Tools were provided to enhance documentation of patient follow-up (use was optional and up to the discretion of facilities).

The training of health facility and/or ART clinic in-charges (approximately 2 senior level staff per facility) and district staff (approximately 1–2 per district) was a two-day training in Kampala (approximately 60 participants total) using didactic sessions with slides as well as small group discussions. This was considered a training of trainers: Participants were given a trainer’s manual to be used at the facility trainings. The training of expert clients was a one-day training at each facility led by the ART in-charge, study staff, and where possible, a representative from the Ministry of Health, and expert clients were given flip charts as reference materials. The training materials were developed and adapted from a number of sources including an elimination of mother-to-child transmission training curriculum previously developed by the Uganda Ministry of Health, materials used in a small pilot led by the Uganda Ministry of Health and the Clinton Health Access Initiative in pediatric patients only, a flipchart for patient education developed by the World Health Organization (https://www.who.int/hiv/pub/imai/patient_flipchart/en/), and discussions with Ministry of Health, healthcare workers, and training experts. Best practices promoted in the trainings included improved tracking and documentation of patient appointments, contact information, and follow-up attempts, conducting up to two phone calls and two home visits to patients who missed appointments or did not link to care, and strengthened patient group education and individual counseling services to emphasize the importance of staying in care.

### Study design

The intervention was evaluated using a pre−/post-intervention study design comparing patient outcomes before and after implementation, clustered by intervention facilities. Data from the 9 months prior to rollout of the intervention (December 25, 2015-September 25, 2016) was retrospectively collected in September–October 2016. The trainings were conducted in October–November 2016 and the intervention was considered to be in place as of November 14, 2016. Facility monitoring visits were conducted monthly for the first 3 months of intervention implementation and every other month for the following months to assess degree of implementation and troubleshoot any issues. Data from the 9 months after rollout of the intervention (November 14, 2016-August 14, 2017) was retrospectively collected in August 2017. Facility registers (HIV counseling and treatment register, daily lab testing log, early infant diagnosis (EID) register for polymerase chain reaction (PCR) testing, pre-ART register, ART register) and HIV patient care cards were examined to compare outcomes at baseline and endline. At baseline, we noted several pieces of information that were not part of the data collection plans in the original study protocol but that were available in the data sources and would be useful to better describe the study population: Entry point, history of HIV tests, TB status, and marital status for individuals testing positive for HIV and time since diagnosis, CD4 count and stage, and pregnancy/lactation status among individuals initiating treatment; therefore, we obtained approvals from the IRBs overseeing this study to collect this additional information at endline.

### Primary outcomes

One of the primary study outcomes was linkage to care, defined as patients newly diagnosed with HIV who registered in pre-ART or ART care at the facility within 1 month of diagnosis. Adult patients who were diagnosed between December 25, 2015 and June 25, 2016 were sampled for inclusion in the pre-intervention period of the study and all pediatric patients diagnosed in that timeframe were included [21]. Due to budgetary constraints and for efficiency purposes given the large number of newly initiated patients, we did not collect data on all newly diagnosed patients but rather systematically sampled every xth patient on the testing register such that a minimum of 40 patients were sampled per facility and the smallest possible number of patients beyond that were sampled. For example, if there were 80 patients on the register, every other patient on the register was sampled. If the number of patients testing during the relevant time period was less than 60 patients, all patients were sampled for simplicity. Similarly, newly diagnosed adults were sampled and all newly diagnosed pediatric patients were included in the post-intervention period from November 14, 2016 and May 14, 2017. Newly diagnosed patients were followed for 3 months to assess linkage, and secondary analyses assessed linkage to care within 1 day, 1 week, or 3 months of HIV diagnosis.

The second primary study outcome was retention in care, defined as patients newly initiated on ART who had at least 1 visit to the health facility for ART care 3–6 months after initiation; this matches the Uganda Ministry of Health’s definition of retention. Because the retention outcome required 6 months of follow-up out of the 9-month study periods, all patients who initiated ART at study facilities between December 25, 2015 and March 25, 2016 and were listed on the ART register were included in the pre-intervention retention assessment; patients initiating between November 14, 2016 and February 14, 2017 were included in the post-intervention retention assessment. Patient data were collected for the 6 months after ART initiation, and secondary analyses assessed the proportion of patients attending a first ART follow-up appointment, at least 4, 5, or 6 appointments over 6 months, mean number of appointments per patient, and adherence to appointment visit schedule (defined as coming to the facility within 1 week of the scheduled appointment). Patients missing the optimal data source for appointment dates (the patient care card) were excluded from the analysis on adherence to appointment schedule, as other data sources such as the ART register had less precision in reporting the appointment dates and often only reported appointment month.

### Data analysis

Patient characteristics were assessed using proportions and medians/interquartile ranges (IQR). For the primary analyses comparing dichotomous linkage and retention outcomes before versus after implementation of the intervention, univariable logistic regression was performed. Linear regression was performed for continuous outcomes. Where univariable analyses were statistically significant or borderline significant, multivariable analyses were conducted adjusting for patient age group and sex. Facility-level clustering was accounted for in all individual-level regression analyses using the vce(cluster) function in Stata and the missing indicator method was used to account for missing covariate data.

Given that the intervention may have taken weeks to months to ramp up fully after the initial training, linkage and retention were examined by intervention month and sensitivity analyses were conducted in which the first month or months of the intervention were excluded from the analysis as a “ramp-up” period.

Analyses were conducted across all ages and stratified by age group. Facility-level characteristics were examined in relation to change in linkage by collapsing linkage and retention data to facility averages before versus after the intervention was implemented and performing linear regression with post-intervention linkage/retention as the outcome and facility characteristics such as size, staffing levels, and funding as the predictor variable, adjusting for pre-intervention linkage/retention. The study team also independently developed a facility leadership and participation score based on observations made at study monitoring visits. ART in-charge leadership and participation were each given a score of 0–2 (with higher scores indicating better leadership and participation) and expert clients/facilitators were also given scores of 0–2 in leadership and participation. These individual scores were summed for a combined maximum score of 8 and were assessed in relation to linkage and retention.

All analyses were completed in StataSE 13 (College Station, Texas).

### Costing

Program costs were calculated, including the cost of the trainings, facility funding for phone airtime and home visits, and program supervision, and used to estimate the cost per additional patient linked to care and cost per additional patient retained in care by dividing the cost over a defined period to the number of additional patients linked to and retained in care over that timeframe using study outcome data.

## Results

### Linkage to care

The study sample to assess linkage to care initially included 1945 patients. 2 patients were excluded from the sample because they died over follow-up and 43 were excluded because they were reported as transferring to another facility over follow-up and this information could not be corroborated (these patients were included in the dataset in sensitivity analyses to ensure that findings were consistent). The final sample included 1900 patients newly diagnosed with HIV (928 pre-intervention, 972 post-intervention).

There were not substantial differences in demographic characteristics of patients in the pre-intervention sample versus post-intervention sample, although data on certain patient characteristics were collected post-intervention only (Table 1). Overall, 60.2% of patients were female. 7.2% of patients were age < 10 years and 8.6% were 10–18 years. Among patients 10–18 years, sex demographics were more skewed: only 20.9% of those patients were male. While most patients (59.0%) were missing information on clinical stage at diagnosis, few patients with known stage were stage III-IV (6.0%). Among post-intervention patients, most (80.5%) were identified through facility-based non-prevention of mother-to-child transmission (PMTCT) testing; 7.1% came from PMTCT entry points and 10.1% came through patient outreach testing. 38.3% of patients were receiving their first HIV test, while 43.0% of patients were receiving their third HIV test or greater in the last year. 60.0% of the children < 10 years and 55.4% of adolescents 10–18 years were being tested for the first time. 3.4% of patients were known tuberculosis (TB) positive. There was a relatively high proportion of missing data on several demographic factors due to this information not being routinely documented at some facilities.

**Table 1:** Characteristics of 1900 patients testing HIV-positive pre- versus post-intervention at 20 facilities in Uganda^a^

Characteristic	Pre-intervention		Post-intervention	
	*N*	%	*N*	%
Total	928		972	
Sex				
Female	558	60.1%	586	60.3%
Male	370	39.9%	386	39.7%
Age group				
< 10 years	72	7.8%	65	6.7%
10–18 years	80	8.6%	83	8.5%
19–48 years	683	73.6%	729	75.0%
49+ years	71	7.7%	59	6.1%
Missing	22	2.4%	36	3.7%
Clinical stage at diagnosis				
I	290	31.3%	296	30.5%
II	90	9.7%	56	5.8%
III	19	2.1%	17	1.8%
IV	6	0.7%	5	0.5%
Missing	523	56.4%	598	61.5%
Entry point				
PMTCT	–	–	69	7.1%
Facility-based non-PMTCT	–	–	782	80.5%
Outreach	–	–	98	10.1%
Missing	–	–	23	2.4%
First HIV test				
Yes	–	–	372	38.3%
No	–	–	567	58.3%
Missing	–	–	33	3.4%
Third HIV test or greater in last year				
Yes	–	–	418	43.0%
No	–	–	514	52.9%
Missing	–	–	40	4.1%
TB status				
Positive			33	3.4%
Negative or unknown			939	96.6%
Marital status				
Married	–	–	428	44.0%
Never married	–	–	269	27.7%
Separated, divorced, or widowed	–	–	104	10.7%
Cohabitating	–	–	32	3.3%
Missing	–	–	139	14.3%

^a^2 patients were excluded from the original sample because they died over follow-up; 43 were excluded because they were reported as transferring to another facility over follow-up and this information could not be corroborated. Data for certain indicators were only collected at endline

There were small increases in linkage to care after implementation of the intervention (Table 2; e.g. linkage to care within 1 month: 52.9% pre-intervention versus 54.9% post-intervention), but increases were not statistically significant regardless of the timeframe assessed (e.g. adjusted odds ratio, aOR for linkage within 1 month: 1.09, 95% confidence interval, CI: 0.78–1.53; *p* = 0.63). Linkage to care did not change substantially over the intervention timeframe and there were not statistically significant increases in linkage in analyses stratified by sex or age group.

**Table 2:** Linkage to care among 1900 patients testing HIV-positive pre- versus post-intervention^a^

Outcome	Pre-intervention		Post-intervention		Univariable OR (95% CI)	*p*	Multivariable aOR (95% CI)	*p*
	*N*	%	*N*	%				
Total	928		972					
Same day linkage to care	271	29.2%	303	31.2%	1.10 (0.81–1.50)	0.59	1.09 (0.80–1.49)	0.57
Linkage to care within one week	432	46.6%	472	48.6%	1.08 (0.78–1.50)	0.63	1.08 (0.79–1.49)	0.62
Linkage to care within 1 month	491	52.9%	534	54.9%	1.09 (0.77–1.54)	0.65	1.09 (0.78–1.53)	0.63
Linkage to care within 3 months	516	55.6%	562	57.8%	1.09 (0.76–1.57)	0.63	1.10 (0.77–1.57)	0.60

^a^Univariable and multivariable logistic regression accounting for facility-level clustering. Multivariable models adjust for patient age and sex

However, change in linkage to care after implementation of the intervention varied greatly by facility and several facility-level characteristics predicted greater increases in linkage to care. Overall, there was a greater increase in linkage among smaller facilities/programs: level III facilities (13.4%) compared to level IV facilities (− 1.6%; effect estimate: 14.2, 95% CI: 0.5–27.9%; *p* = 0.04); facilities with fewer expert clients (< 5: 11.5%; 5+: − 4.4%; effect estimate: 14.3, 95% CI: 3.3–28.3%; *p* = 0.045); and facilities with fewer adult ART days per week (1 day: 16.7%; 2+ days: − 4.3%; effect estimate: 17.4, 95% CI: 3.9–30.9%; *p* = 0.01). There was also a greater increase in linkage among facilities reporting issues with phone funding at baseline (16.0%) compared to those who did not report this as an issue (− 0.1%; effect estimate: 15.8, 95% CI: 1.8–29.8%; *p* = 0.03).

### Retention in care

Initially, 1442 patients who initiated on ART within the specified time frame were included in the study sample. However, 28 patients were excluded from the sample because they died over the 6 months of study follow-up and 58 were excluded because they were reported as transferring to another facility over follow-up and this information could not be corroborated (although these patients were included in the dataset in sensitivity analyses to ensure that findings were consistent). The final sample included 1356 patients newly initiated on ART (678 pre-intervention, 678 post-intervention).

Overall, 63.0% of patients newly initiated on ART were female; 4.2% were age less than 10 years and 4.1% were age 10–18 years (Table 3). Again, among patients 10–18 years, sex demographics were skewed: only 18.2% of those patients were male. Among patients from the post-intervention period, 39.4% initiated ART within 1 month of diagnosis and 6.3% initiated at least 1 year after diagnosis (note that Test and Treat policies were not implemented universally until 2017 in Uganda, when all patients became eligible for ART initiation regardless of CD4 T-cell count; however, some facilities with high-risk patients started Test and Treat prior to 2017). 5.1% of patients were stage III or IV at initiation and 6.3% of patients were missing clinical stage at initiation, while 15.6% of patients had a CD4 count < 350 cells/mcL at initiation and 66.4% had missing CD4 information. Finally, 19.0% of patients were pregnant at initiation. There was a relatively high proportion of missing data on several demographic factors due to this information not being routinely documented at some facilities.

**Table 3:** Characteristics of 1356 patients initiating antiretroviral therapy pre- versus post-intervention^a^

Characteristic	Pre-intervention		Post-intervention	
	*N*	%	*N*	%
Total	678		678	
Sex				
Female	408	60.2%	446	65.8%
Male	270	39.8%	232	34.2%
Age group				
< 10 years	30	4.4%	27	4.0%
10–18 years	20	3.0%	35	5.2%
19–48 years	542	79.9%	558	82.3%
49+ years	64	9.4%	54	8.0%
Missing	22	3.2%	4	0.6%
Time from diagnosis to ART initiation				
< 1 month	–	–	267	39.4%
1- < 3 months	–	–	49	7.2%
3- < 6 months	–	–	28	4.1%
6- < 12 months	–	–	26	3.8%
12+ months	–	–	43	6.3%
Missing	–	–	265	39.1%
Clinical stage at initiation				
I	–	–	432	63.7%
II	–	–	168	24.8%
III	–	–	30	4.4%
IV	–	–	5	0.7%
Missing	–	–	43	6.3%
CD4 count at initiation				
< 200 cells/mcL	–	–	36	5.3%
200- < 350 cells/mcL	–	–	70	10.3%
350- < 500 cells/mcL	–	–	73	10.8%
500+ cells/mcL	–	–	49	7.2%
Missing	–	–	450	66.4%
Pregnancy/lactation status at initiation				
Pregnant	–	–	129	19.0%
Lactating	–	–	15	2.2%
None	–	–	534	78.8%

^a^28 patients were excluded from the original sample because they died over the 6 months of follow-up; 59 were excluded because they were reported as transferring to another facility over follow-up and this information could not be corroborated. Data for certain indicators were only collected at endline

Patient appointment attendance appeared to increase after implementation of the intervention in univariable models as well as models adjusted for patient age and sex (Table 4). The percentage of patients coming to their first appointment increased from 85.3 to 90.3% (aOR: 1.61, 95% CI: 1.05–2.47, *p* = 0.03) and the percentage of patients coming to at least 4 appointments over 6 months of follow-up increased from 58.0 to 67.1% (aOR: 1.50, 95% CI: 1.11–2.04, *p* = 0.01). Overall, the mean number of patient appointments increased after the intervention from 3.7 to 4.3 (effect estimate: 0.67, 95% CI: 0.29–1.04, *p* = 0.002) and more patients (44.5% pre-intervention versus 55.2% post-intervention) adhered to their appointment schedule (aOR: 1.58, 1.13–2.22, *p* = 0.01). In models using the full data set, the increase in MOH-defined retention (coming to at least one appointment 3–6 months after ART initiation) was not statistically significant (71.7% versus 75.7%, aOR: 1.25, 95% CI: 1.25, 95% CI: 0.94–1.67, *p* = 0.12). However, in sensitivity analyses, we noted that patient retention increased each month over the 3-month study enrollment period after the intervention was implemented. If the first month of intervention implementation was considered a “ramp-up” period and those data were excluded, the increase in retention before versus after the intervention became statistically significant (71.7% versus 77.6%, aOR: 1.41, 95% CI: 1.08–1.84, *p* = 0.01).

**Table 4:** Retention in care and adherence to appointment schedule among 1356 patients initiating antiretroviral therapy pre- versus post-intervention^a^

Outcome	Pre-intervention		Post-intervention		Univariable OR/Effect estimate (95% CI)	*p*	Multivariable aOR/Effect estimate (95% CI)	*p*
	*N*	%/Mean (SD)	*N*	%/Mean (SD)				
Total	678		678					
Came to first appointment	578	85.3%	612	90.3%	1.60 (1.04–2.47)	0.03	1.61 (1.05–2.47)	0.03
Came to at least 4 appointments	393	58.0%	455	67.1%	1.48 (1.10–1.99)	0.01	1.50 (1.11–2.04)	0.01
Mean number of appointments	678	3.7 (2.2)	678	4.3 (2.4)	0.65 (0.28–1.02)	0.002	0.67 (0.29–1.04)	0.002
Adhered to appointment schedule (within 1 week)^a^	236	44.5%	295	55.2%	1.54 (1.10–2.15)	0.01	1.58 (1.13–2.22)	0.01
Came to an appointment 3–6 months after initiation (MOH-defined retention)	486	71.7%	513	75.7%	1.23 (0.92–1.64)	0.17	1.25 (0.94–1.67)	0.12
Came to an appointment 3–6 months after initiation (MOH-defined retention), excluding first month of post-intervention enrollments	486	71.7%	347	77.6%	1.37 (1.06–1.78)	0.02	1.41 (1.08–1.84)	0.01

^a^Univariable and multivariable logistic regression accounting for facility-level clustering. Multivariable models adjust for patient age and sex. Patients missing optimal data source for appointment dates (patient care card) were excluded from this analysis

The increases in patient adherence to appointment schedule and retention in care were more pronounced in pediatric and adolescent patients ages less than 19 years (Table 5). For example, adherence to appointment schedule increased from 33.3 to 60.9% in patients under 19 years (aOR: 3.31, 95% CI: 1.41–7.79, p = 0.01) and retention increased from 64.0 to 83.9% (aOR: 2.92, 95% CI: 1.31–6.51, p = 0.01).

**Table 5:** Retention in care and adherence to appointment schedule pre- versus post-intervention stratified by age group^a^

Outcome	Pre-intervention		Post-intervention		aOR/Effect estimate (95% CI)	*p*
	*N*	%	*N*	%		
< 10 years	30		27			
Came to at least 4 appointments	17	56.7%	21	77.8%	2.65 (0.74–9.53)	0.14
Adhered to appointment schedule	9	36.0%	17	85.0%	12.39 (3.64–42.19)	< 0.001
MOH-defined retention	22	73.3%	25	92.6%	4.36 (0.94–20.20)	0.06
10–18 years	20		35			
Came to at least 4 appointments	11	55.0%	23	65.7%	1.41 (0.49–4.04)	0.52
Adhered to appointment schedule	4	28.6%	11	42.3%	2.01 (0.52–7.71)	0.31
MOH-defined retention	10	50.0%	27	77.1%	3.43 (1.19–9.83)	0.02
19+ years	606		612			
Came to at least 4 appointments	353	58.3%	408	66.7%	1.44 (1.05–1.97)	0.02
Adhered to appointment schedule	218	45.7%	265	54.8%	1.46 (1.03–2.06)	0.03
MOH-defined retention	440	72.6%	458	74.8%	1.14 (0.84–1.56)	0.40
< 19 years	50		62			
Came to at least 4 appointments	28	56.0%	44	71.0%	1.95 (0.83–4.59)	0.13
Adhered to appointment schedule	13	33.3%	28	60.9%	3.31 (1.41–7.79)	0.01
MOH-defined retention	32	64.0%	52	83.9%	2.92 (1.31–6.51)	0.01

^a^Sex-adjusted logistic regression accounting for facility-level clustering

Greater increases in retention were observed in facilities that demonstrated a stronger leadership and participation score (mean increase of 9.3% in facilities scoring 4–8 versus mean decrease of 5.5% at facilities with a score of < 4; effect estimate: 16.1, 95% CI: 5.5–26.7%; p = 0.01).

The annual cost per additional patient retained in care was estimated to be $47, dropping to $32 if data from the one-month intervention ramp-up period were excluded.

## Discussions

In this pre−/post-intervention study spanning 18 months, we observed a statistically significant increase in patient appointment attendance after implementation of a management intervention to promote improved patient follow-up and enhanced counseling. After excluding data from the intervention “ramp-up” period, there was a statistically significant increase in patient retention in care as well. Increases in patient retention and adherence to appointment schedule were most dramatic in pediatric and adolescent patients, who had a greater gap in retention at baseline compared to adults. Greater increases in retention in care were observed in facilities that demonstrated stronger leadership and participation in the intervention. We did not observe a significant increase in patient linkage to care, although there were greater improvements in patient linkage to care among smaller programs and in facilities reporting inadequate funding for phone follow-up at baseline.

Our study findings around improved retention after implementation of this intervention are in line with recent systematic reviews concluding that strategies such as task-shifting to peer health worker care as well as adherence support through counseling and education were effective in improving retention [15–17]. A recent study among 5781 patients in Uganda, Kenya, and Tanzania reported that better patient tracing and follow-up procedures increased patient reengagement in care [22]. Our findings also complement a smaller study in Uganda with similar intervention components: After implementation of an appointment system, patient appointment books, and differentiated care for stable patients in 6 poorly-performing district hospitals, the proportion of missed appointments and medication gaps decreased; in this study, the reduction in medication gaps was greater in newly initiated patients [23]. A quasi-experimental study in 12 hospitals in Kenya also found that an intervention involving better appointment tracking at the facility and more supervision led to better patient appointment attendance [24].

We observed a greater increase in retention among pediatric and adolescent patients, although this subgroup was small. Pediatric and adolescent patients tend to have lower retention compared to adults, so this represents a high-risk group that may need additional support. A recent systematic review on strategies to increase linkage and retention noted that there is a paucity of data on effective interventions for adolescents [17], and the findings from this study provide much-needed evidence on interventions that may be effective in this age group. Our findings support the implementation of this relatively low-cost and scalable intervention to real-world settings struggling with retention.

The lack of a significant improvement in linkage overall may be explained by a number of factors. First, there were a high proportion of migratory patients at many of the study facilities, with no contact information or fixed address for follow-up. When these patients tested positive, facility staff had no means to reach them again if they did not immediately enroll in care. Second, the laboratory staff conducting HIV testing did not necessarily attend the intervention training, and testing services exist across many entry points within facilities (such as antenatal care, voluntary counseling and testing, and outreach), and patient information for those who tested positive at times was not collected or did not make it to the ART clinic for staff to track and follow-up. Based on this, we recommend that HIV testing registers are updated to include space for detailed patient contact information and that trainings on patient follow-up include all staff involved in patient testing, treatment, documentation, follow-up, and counseling to ensure that everyone works together to improve the process. With better implementation of these practices, it may be possible to improve linkage to care in certain settings. For example, in a randomized trial in Uganda, an extended counseling program involving a post-test visit and monthly 2-hour home visits nearly doubled linkage to care (67.5% versus 38.5%) [25].

This study had several limitations. Given the pre−/post-study design, we were unable to account for temporal changes in the study outcomes due to policy changes or other factors. The analysis relies on retrospective collection of data using health records that in some cases may have been incomplete/incorrect. We were unable to confirm outcomes of patients who were lost to follow-up; some of these patients may have transferred to another facility for care or died. The accuracy and completeness of the registers may have improved because of the intervention, affecting the comparisons; however, no substantive changes occurred in the register system after the intervention. Study results may not be generalizable to sites with much higher baseline retention values and the impact of the intervention may be attenuated in these settings. Finally, we did not assess the sustainability of the intervention over a longer time period.

Strengths of this study include the relatively large number of facilities and large number of patients (> 3000), which both demonstrate a wide variety of characteristics; the inclusion of pediatric and adolescent patients, which are priority groups due to higher risk of attrition at these ages; and the comprehensive data collection and analysis accounting for patient- and facility-level characteristics and including qualitative feedback from facility staff and patients.

## Conclusion

Given the success of this intervention in improving patient retention in care, particularly for pediatric and adolescent patients, and its relatively low cost, this approach should be considered for scale-up in similar settings in Uganda and elsewhere. A recent analysis noted that interventions to improve linkage and retention in care were likely to be more cost-effective than increasing HIV testing rates [26]; these types of relatively low-cost interventions should be emphasized as a strategy to reduce the burden of HIV.

## Abbreviations

ART: Antiretroviral therapy
aOR: Adjusted odds ratio
DHIS: District Health Information System
EID: Early infant diagnosis
IQR: Interquartile range
PCR: Polymerase chain reaction
PLWHIV: People living with HIV
PMTCT: Non-prevention of mother-to-child transmission
TB: Tuberculosis
